# Bcr-Abl tyrosine kinase inhibitors- current status

**DOI:** 10.1186/1750-9378-8-23

**Published:** 2013-06-20

**Authors:** Anum Mughal, Hafiz Muhammad Aslam, Aga Muhammad Hammad Khan, Shafaq Saleem, Ribak Umah, Maria Saleem

**Affiliations:** 1 Dow Medical College, Dow University of Health Sciences, Karachi, Pakistan; 2 Sindh Medical College, Dow University of Health Sciences, Karachi, Pakistan; 3 Final Year Student Of Dinajpur Medical College, Dakshin Dinajpur District, West Bengal, Bangladesh; 4 First Year Student of Karachi Medical And Dental College, Karachi University, Karachi, Pakistan

## Abstract

Bcr-Abl plays a central role in the development of chromosome positive leukaemia. Chronic Myeloid leukaemia occurs due to increase proliferation and resistance to apoptosis by Bcr-Abl positive cells. Imatinib (STI571) is the first drug in the family of Bcr-Abl tyrosine kinase inhibitors while Nilotinib (AMN107) and Dasatinib (BMS-345825) are second generation drugs that are intended to have less resistance and intolerance than imatinib. Ponatinib (AP24534) an orally active Bcr-Abl Tyrosine Kinase Inhibitor and Bafetinib (INNO-406) have efficacy against various point mutations in the Bcr-Abl kinase. 1, 3, 4 thiadiazole derivatives has also displayed moderate inhibitory action on both Abl and Src kinase family. However there are varieties of Bcr-Abl inhibitors but Nilotinib is still the frontline tyrosine kinase inhibitors.

## Letter to editor

The Bcr-Abl chimeric protein is thought to play a central role in the pathogenesis of Philadelphia (Ph) chromosome-positive leukaemia, notably Chronic Myeloid Leukaemia (CML) [1]. This abnormality was discovered by Janet Rowley in 1972 and it is due to the reciprocal translocation between chromosome 9 and 22. Three fusion proteins can be formed as a result of breakpoint in Bcr, all of which exhibit deregulated PTK activity [2-4]. Basic mechanisms that have been attributed to Bcr-Abl positive cells, particularly in CML, are increased proliferation, increased resistance to apoptosis [5-7], and an alteration of their adhesion properties [8,9]. Mutational analysis show that the Tyrosine Kinase activity of the protein is an absolute requirement for malignant transformation, and that it cannot be complemented by any downstream effectors [10,11]. For these reasons, an inhibitor of the Bcr-Abl tyrosine kinase should be an effective and selective treatment for CML.

Selective therapies are aimed for the treatment of CML because its target is well defined in contrast to other cancers of body [12]. Hundreds of protein kinases are known in human genome and a drug was required that targeted a single ATP binding site of protein kinase [13]. By blocking the binding of ATP, phosphorylation is prevented and Bcr- Abl expressing cells either have a growth disadvantage or they undergo apoptosis [7].

Imatinib (STI571) is the first drug of Bcr-Abl tyrosine kinase inhibitors that prevents ATP from binding by itself binding to Abl domain via six hydrogen bond interactions [14]. Hydrogen bonds involve the pyridine-N and backbone-NH of Met-318, the aminopyrimidine and side chain hydroxyl of Thr-315, the amide-NH and side chain carboxylate of Glu-285, the carbonyl and backbone-NH of Asp-381, the protonated methylpiperazine with the backbone-carbonyl atoms of Ile-360 and His-361. Additionally, a number of van der Waals interactions contribute to binding [13-15]. Resistance faced by imaitinab can be subdivided into BCR independent and dependant mechanisms [16]. Dependant mechanism depend upon the duplication of BCR-ABL tyrosine kinase gene in DNA sequence leading to higher expression of pathogens [12]. Point mutation in the kinase domain of Bcr-Abl leading to disrupt in the binding site of imatinib on the tyrosine kinase, resulting in the loss of sensitivity of drug [16]. The T315I is a unique mutation because of its resistance to all approved Bcr-Abl inhibitors, prior to ponatinib [17]. It may be due to the displacement of cytosine to thiamine (C->T) base pair at 944 of the Abl gene. It cause the elimination of critical O2 molecule needed for hydrogen bonding between imatinab and Bcr-Abl kinases [12]. Most common mutation has been occurred in ATP binding and activation loop. It cause the derangement of loops as a result of which kinase domain cannot assume inactive conformation required for imatinib binding [16]. Bcr independent resistance occur either due to over expression of P-glycoprotein efflux pump, activation of Src family kinase or may be because of low expression, activity or polymorphism of OCT1 [12,18]. Solution for combating resistance is to increase the dose of imitinab, administration of multiple Abl kinase inhibitors and usage of two drugs simultaneously who have different pathways [16,19]. Nilotinib (AMN107) and Dasatinib (BMS-345825) are second generation drugs that are intended to have less resistance and intolerance than Imatinib [12]. Nilotinib is a selective inhibitor and binds to the inactive conformation of the Abl kinase domain, largely through lipophilic interactions and thus blocks its catalytic activity, being 10–30 fold potent than Imatinib [19,20]. Nilotinib binds to kinase domain with the help of H2 bond interaction involving pyridyl-N and backbone of NH of Met-318, amino NH and side chain of OH of Thr 315, amido NH, side chain carboxylate of Glu-286 and amido carbonyl with backbone NH of Asp −381 [21,22]. It is effective against all type of resistances except T315I mutation. Its failure against T315I is due to the loss of an H-bond interaction between threonine-O and aniline-NH on nilotinib and a steric clash between the isoleucine-methyl group and 2-methylphenyl phenyl group of nilotinib [19-21]. Dasatinib is multi targeted inhibitor of wild type Bcr-Abl and Src family kinases having additional inhibitory activity against downstream kinases [23]. Contrary to most Tyrosine Kinase Inhibitors, Dasatinib bind to active conformation of Abl kinase [15]. First and second generations inhibitors have provided promising results but new mutations are continuously being encountered that requires discovery of more drugs.

Bosutinib is based on a quinolone scaffold and is related to AstraZeneca quinazoline template and it also had the ability of inhibiting mutation of T3151 [20]. Ponatinib (AP24534) an orally active Bcr-Abl Tyrosine Kinase Inhibitor effective against the T315I mutation had been approved for a phase II clinical trial [24]. Bafetinib (INNO-406) with efficacy against various point mutations in the Bcr-Abl kinase, with fewer adverse effects and with narrower kinase spectra, is also in phase II clinical trials [25]. Befitinib and Imatinib has structural and binding similarities, the notable difference being hydrophobic interaction between the trifluoromethyl group and the hydrophobic pocket created by Ile-293, Leu-298, Leu-354, and Val-379 [26]. 1, 3, 4 thiadiazole derivatives has also displayed moderate inhibitory action on both Abl and Src kinases with similar binding properties as dasatinib, and are still in trials to prove a novel way to inhibit Tyrosine Kinases [27].

Although there are more potent Bcr-Abl Tyrosine Kinase Inhibitors are available, but Imatinib still remains the frontline Tyrosine Kinase Inhibitors. Nilotinib, Dasatinib, Bosutinib and Ponatinib are approved for the treatment of Imatinib resistant or intolerant CML. The availability of highly potent tyrosine kinase inhibitors, such as nilotinib, has broadened the treatment armamentarium in CML. Nilotinib appears to overcome imatinib resistance in patients with chronic, accelerated, and blastic phase CML, producing sustained cytogenetic and haematological responses [28]. The first line data for these drugs are encouraging and suggest that some or all of them may replace Imatinib as a frontline standard Bcr- Abl tyrosine kinase inhibitor in the near future.

## Competing interests

Authors declare they have no competing interest.

## Authors’ contributions

AM and HMA did manuscript drafting, AMHK, SS, RU and MS did critical review. All have given final approval of the version to be published.
